# Retraction: A Regulatory Mechanism Involving TBP-1/Tat-Binding Protein 1 and Akt/PKB in the Control of Cell Proliferation

**DOI:** 10.1371/journal.pone.0222287

**Published:** 2019-09-04

**Authors:** The PLOS ONE Editors

Following publication of this article [1], the following concerns were raised:

- Fig 1A actin panel lane 4 appears similar to lane 7;
- Fig 5B actin panels look similar to each other;
- Fig 5B upper pAkt panel, lanes 1, 5, and 6 appear to have a background different to the other lanes;
- Fig 5F Akt panel lanes 1–6 appear similar to Fig 6B C8 panel lanes 2–7 and to Fig 6E Akt panel lanes 1–6;
- Fig 6A C8 lanes 2, 3 appear similar to lanes 5, 6 and there are vertical discontinuities after lane 4;
- Fig 6B pAkt panel contains background discontinuities across lanes 1–4;
- Fig 6B TBP-1 panel contains background discontinuities after lane 4 such that the background differs between lanes 1–4 and 5–7;
- Fig 6C pAkt panel contains background discontinuities between lanes 2 and 3.

The authors provided data underlying replication experiments that confirm some of the results presented in the article. However, owing to the length of time since the experiments were carried out, none of the original underlying data are available to resolve the concerns raised.

In light of the unresolved concerns that question the validity of the study’s findings, the *PLOS ONE* Editors retract the article.

AP notified the journal office that DA, PR, VC, VdF, GLM, AP agree to the retraction, but are confident in the findings and stand by the published results. MS, LF, FT, LB, LS did not respond to the retraction notification.
